# Supplementary material for: IGF-1 impacts neocortical interneuron connectivity in epileptic spasm generation and resolution
Source: Neurotherapeutics. 2024 Nov 8;22(1):e00477. doi: 10.1016/j.neurot.2024.e00477 (PMC11743118; doi:10.1016/j.neurot.2024.e00477)
Supplement: Multimedia component 1 — Supplementary Table 1. Summary of Resected Surgery Patients’ Neuropathological Findings, Seizure Classification and Type of Surgery Undertaken. Patient ID: S- infantile spasms patient number, C- Tumor control patient number. [file mmc1.docx]

| ID | Sex | Pathology | Seizure (SZ) type | Age at surgery  (yrs) | Surgery type |
| --- | --- | --- | --- | --- | --- |
| S1 | F | Intrauterine stroke,  intraventricular hemorrhage, refractory epilepsy with cluster seizures | Infantile Spasms at 6 months of age - later DEVELOPED FOCAL motor sZ | 3 | Right hemispherectomy |
| S2 | F | Down syndrome, perinatal stroke, refractory infantile spasms | infantile spasms at 6 months  Spasms persisted  until surgery | 3 | Left  hemispherectomy |
| S3 | M | Grade 4 left Intraventricular hemorrhage  Refactory Epilepsy | infantile spasms at 8 months - later developed focal motor sZ | 1.75 | Left hemispherectomy |
| S4 | M | Vein of Galen malformation and right-hemispheric arteriovenous malformation, refractory spasms | infantile spasms at 6 months; Spasms persisted  until sugery | 4 | Right hemispherectomy |
| C1 | M | glioma with mixed oligodenroglioma, angiocentric glioma and dysembryoplastic neuroepithelial tumor | Altered awareness, focal motor sZ prior and leading to tumor diagnosis | 6 | Left anterior temporal Glioma resection |
| C2 | F | ANGIOCENTRIC GLIOMA, | focal motor SZ, prior and leading to tumor diagnosis | 8 | left temporal angiocentric glioma resection |
| C3 | F | DYSEMBRYOPLASTIC  NEUROEPITHELIAL TUMOR (DNET) | focal motor SZ, prior and leading to tumor diagnosis | 5 | Right anterior medial temporal DNET resection |
| C4 | M | GANGLIOGLIOMA, | focal motor SZ, prior and leading to tumor diagnosis | 12 | Right anterior temporal Ganglioglioma resection |
